# Foreign Travel and Decreased Ciprofloxacin Susceptibility in *Salmonella enterica* Infections

**DOI:** 10.3201/eid1701.100999

**Published:** 2011-01

**Authors:** Manar Al-Mashhadani, Robert Hewson, Roberto Vivancos, Alex Keenan, Nick J. Beeching, John Wain, Christopher M. Parry

**Affiliations:** Author affiliations: University of Liverpool, Liverpool, UK (M. Al-Mashhadani, R. Hewson, C.M. Parry);; Health Protection Agency Moorgate Point, Liverpool (R. Vivancos, A. Keenan);; University of Manchester, Manchester, UK (R. Vivancos);; Liverpool School of Tropical Medicine, Liverpool (N.J. Beeching);; Health Protection Agency Colindale, London, UK (J. Wain)

**Keywords:** Salmonella, nontyphoidal Salmonella enterica, bacteria, antimicrobial drug resistance, ciprofloxacin, nalidixic acid, foreign travel, dispatch

## Abstract

To determine antimicrobial drug resistance patterns, we characterized nontyphoidal *Salmonella enterica* strains isolated in Liverpool, UK, January 2003 through December 2009. Decreased susceptibility to ciprofloxacin was found in 103 (20.9%) of 492 isolates. The lower susceptibility was associated with ciprofloxacin treatment failures and with particular serovars and phage types often acquired during foreign travel.

Nontyphoidal *Salmonella enterica* (NTS) isolates produce a common food-related infection that causes mild and self-limiting diarrhea and, occasionally, a potentially fatal invasive disease with bacteremia and endovascular infection (*1*). Antimicrobial drug therapy, often with the fluoroquinolone ciprofloxacin, is required for treating invasive NTS infections and severe diarrhea in immunocompromised patients. Full resistance to fluoroquinolones is relatively uncommon for NTS infections, but decreased susceptibility to ciprofloxacin (DCS), defined as MIC 0.1–1.0 µg/mL, has become common (*2*). Resistance to nalidixic acid is often used as a marker for DCS, although the validity of this assumption has been debated (*3*), and some have suggested that the ciprofloxacin disk susceptibility zone size breakpoints should be changed to detect such strains (*2*).

DCS is associated with ciprofloxacin treatment failures in patients with typhoid fever (*4*) and probably with invasive NTS infection, although the clinical significance of DCS in NTS has not been widely explored (*5**–**7*). Clinical clues to the presence of DCS in *S. enterica* infections could guide early empirical prescription of antimicrobial drug therapy. Links between DCS and foreign travel have been suggested in reports from Denmark (*8*) and Finland (*9**,**10*). In this study, we characterized the resistance patterns of NTS strains isolated in Liverpool and explored the value of nalidixic acid–resistance testing, the clinical significance of DCS, and possible links between DCS and foreign travel.

## The Study

We studied all isolates of NTS detected in samples submitted to the microbiology department at the Royal Liverpool University Hospital from patients in the Merseyside area from January 2003 through December 2009**.** Isolates were identified by standard biochemical and serologic methods. Disk susceptibility testing was performed for ampicillin, trimethoprim, ciprofloxacin, and ceftriaxone. Nalidixic acid testing was recommended but not consistently performed. The serovar, phage type, and susceptibility of each isolate were confirmed by the Laboratory for Gastrointestinal Pathogens, Health Protection Agency Centre for Infections, Colindale, London. Isolates were stored in glycerol broth at −70°C and later subcultured for determination of MICs to ciprofloxacin and nalidixic acid by using the agar dilution and the Etest strip methods. The control organisms *Escherichia coli* ATCC 25922 and *Staphylococcus* *aureus* ATCC 25923 were used. Breakpoints were defined according to Clinical Laboratory Standards Institute guidelines (*11*). DCS was defined as MIC 0.1–1.0 µg/mL and resistance MIC >1 µg/mL.

Laboratory data, including serovar and antimicrobial drug susceptibility patterns, were merged with statutory notification of diseases data from the Cheshire and Merseyside Health Protection Unit of the Health Protection Agency. This second database contained the travel history (validated through an enhanced questionnaire) of the patients, detailing whether they had a relevant history of foreign travel and, if so, where they had traveled. The medical records of patients with bacteremia were retrieved and reviewed to establish whether ciprofloxacin had been used for therapy and the clinical outcome.

A total of 492 unique NTS patient isolates, including isolates from 116 inpatients, were identified during the study period. Diarrheal isolates accounted for 479 of the total; 11 were from blood, 1 was from a wrist aspirate, and 1 was from a pathology specimen of the large bowel. The number and proportion of isolates resistant to ampicillin were 39 (7.9%), to trimethoprim 22 (4.5%), to ciprofloxacin 2 (0.4%), and to ceftriaxone 6 (1.2%). Resistance to nalidixic acid was determined at isolation for 281 (57.1%) of 492 isolates and was found for 53 (18.9%). When all 492 patient isolates were tested, 103 (20.9%) were resistant to nalidixic acid, including both isolates that were ciprofloxacin resistant. Of 397 (81%) isolates retrievable for MIC testing, all 304 with a MIC of ciprofloxacin <0.1 µg/mL were also susceptible to nalidixic acid, 91 had DCS (0.1–1.0 µg/mL), all but 1 were resistant to nalidixic acid, and 2 were resistant to nalidixic acid and ciprofloxacin with a ciprofloxacin MIC >4.0 µg/mL. Nalidixic acid resistance correlated well with DCS.

Forty-eight named serovars of *S. enterica* were found among the 492 characterized isolates. The serovars most commonly isolated from patients with nalidixic acid resistance were Enteritidis phage type 1 (PT1), Virchow, Newport, and Enteritidis PT21; the first 3 of these were also commonly associated with a history of foreign travel (Table). The higher levels of DCS in serovars Enteritidis and Virchow and lower levels in serovar Typhimurium are consistent with data from Europe, as is the association with particular serovar Enteritidis phage types (*12**,**13*).

**Table:** Relationship between nontyphoidal *Salmonella enterica* serovar, phage type, nalidixic acid resistance, and history of foreign travel, Liverpool, UK, 2003–2009*

Serovar	Total no. (%) patient isolates	No. (%) patients with foreign travel history	No. (%) patients with nalidixic acid–resistant isolate	No. (%) patients with foreign travel history and nalidixic acid–resistant isolate
All	492 (100.0)	110 (22.4)	103 (20.9)	35 (31.8)
Enteritidis PT1	53 (10.8)	16 (30.2)	36 (67.9)	9 (56.3)
Enteritidis PT4	93 (18.9)	12 (12.9)	4 (4.3)	2 (16.7)
Enteritidis PT8	43 (8.7)	8 (18.6)	1 (2.3)	1 (12.5)
Enteritidis PT21	22 (4.5)	5 (22.7)	10 (45.4)	1 (20.0)
Enteritidis (other PT)	104 (21.1)	13 (12.5)	16 (15.4)	3 (23.1)
Typhimurium	44 (8.9)	11 (25.0)	3 (6.8)	1 (9.1)
Newport	14 (2.8)	3 (21.4)	8 (57.1)	3 (100.0)
Virchow	12 (2.4)	9 (75.0)	11 (91.7)	8 (88.9)
Other	107 (21.7)	33 (30.8)	14 (13.1)	7 (21.2)

*PT, phage type.

A relevant foreign travel history was reported for 110 (22.4%) of 492 patients; destinations were identifiable for 105, including 36 countries from across Asia, Europe, South America, and Africa. Countries most commonly implicated were Spain (22; 20.0%), Egypt (11; 10.0%), Turkey (10; 9.1%), India (8; 7.3%), and Thailand (5; 4.6%). Among the 110 isolates from patients who had traveled, 35 (31.8%) were resistant to nalidixic acid compared with 68 (17.8%) from the 382 patients with no history of foreign travel (odds ratio [OR] 2.15, 95% confidence interval [CI] 1.30–3.57; p<0.001). Travel to Egypt (OR 5.3, 95% CI 1.59–17.99; p = 0.007), Spain (OR 3.08, CI 1.27–7.48; p = 0.018), and Thailand (OR 17.51, CI 2.8–109.33; p = 0.002) was associated with DCS; these countries were also identified in other studies (*8**–**10*). High levels of DCS or resistance to ciprofloxacin have been observed among isolates of NTS from Spain and Thailand (*14**,**15*). This study does not include rates of travel to various destinations, so the higher numbers of *S. enterica* isolates with DCS in travelers to some destinations may simply reflect travel patterns.

In a multivariate analysis, after different serovars and phage types and history of foreign travel were adjusted for, DCS was independently associated with serovars Enteritidis PT1 (OR 14.42, CI 6.41–32.43; p<0.001), Enteritidis PT21 (OR 5.81, CI 2.1–16.08; p = 0.001), Newport (OR 9.38, CI 2.8–31.38; p<0.001), and Virchow (OR 62.33, CI 7.37–526.82; p<0.001) but not with a history of foreign travel (OR 1.54, CI 0.82–2.85; p = 0.178). This finding suggests that the association with particular serovars and phage types is greater than any association with foreign travel and that travel is a factor only because travel can facilitate importation of these serovars.

The clinical features of the 11 patients with bacteremia are summarized in Table A1; 10 either were immunosuppressed or had gall bladder disease. Five blood culture isolates were resistant to nalidixic acid with DCS. Ciprofloxacin was the initial drug choice for 3 of the patients infected with a DCS isolate, but in each instance, the drug was changed to an alternative (ceftriaxone for 2 patients, ampicillin for 1) because of an unsatisfactory clinical response. The other 2 patients initially received a cephalosporin, and outcome was acceptable. Although extended-spectrum cephalosporins are the principal alternative antimicrobial drugs for treatment of bacteremic infections, resistance is also emerging (*2**,**13*). Six of the isolates in this study were resistant to ceftriaxone, including 1 of the isolates from a patient with bacteremia, which was susceptible to ciprofloxacin.

## Conclusions

Our data show that one fifth of NTS isolates in Liverpool demonstrated nalidixic acid resistance and that this was a good marker for DCS. The data also suggest that DCS may compromise ciprofloxacin therapy for invasive disease caused by NTS. Infection with particular serovars and phage type, frequently associated with foreign travel, were significant risk factors for infection with an *S. enterica* isolate with DCS, and this information can help guide initial empirical antimicrobial drug choices. Early detection of DCS is essential, but nalidixic acid–resistance testing was not always performed at the time of isolation. Revision of ciprofloxacin disk susceptibility breakpoints would allow such isolates to be detected more easily.

## Appendix

**Table A1:** Clinical features and outcomes for 11 patients with nontyphoidal *Salmonella enterica* bacteremia, Liverpool, UK, 2003–2009*

Patient no.	Strain no.	Serovar and PT	Resistance pattern	Ciprofloxacin MIC, µg/mL	Age, y/ sex	Preexisting condition	Travel	Clinical signs, symptoms	Initial therapy	Outcome
1	31711	Enteritidis PT4	ACSuTTm	<0.060	41/F	New HIV diagnosis, CD4 count 27 x 10^6^ cells/L	No	Fever, cough	Ciprofloxacin	Initial resolution. Subsequent TB diagnosis.
2	33284	Enteritidis PT1	Pansusceptible	<0.060	53/F	Cholecystectomy for stones 2 years previously	No	Fever, complicated pancreatitis	Ciprofloxacin	Empirical ceftriaxone and metronidazole added
3	33474	Enteritidis PT1	NxCp_L_	<0.060	57/F	Previous breast cancer, duodenal ulcer	No	Acute cholecystitis	Cefuroxime/ metronidazole	Empyema, gall bladder disease, pancreatitis
4	43539	Enteritidis PT21	NxCp_L_	0.500	66/M	Aortic aneurysm	Turkey	Fever	Ciprofloxacin	Persistent fever. Ciprofloxacin dose increased and ampicillin added. No evidence of graft infection. Resolution.
5	52612	Enteritidis PT21	Pansusceptible	0.060	25/F	SLE, (mycophenolate and steroid treatment)	Turkey	Fever	Amoxicillin and clavulanic acid	Antimicrobial drug changed to ciprofloxacin. Resolution.
6	52981	Enteritidis PT21	ACCro	0.060	47/F	Post bone marrow allograft	No	Fever	Tazocin and gentamicin	Antimicrobial drug changed to meropenem. Resolution.
7	61246	Newport	NxCp_L_	1.000	75/F	Gall bladder carcinoma Previous isolation of salmonellae from gall bladder, DM, steroids	No	Fever	Ciprofloxacin	Recurrent bacteremia. Antimicrobial changed to ceftriaxone. Eventual resolution.
8	62856	Virchow	ASuTTmNxCp_L_	0.250	34/F	None	Kenya	Fever, abdominal pain	Ceftriaxone	Resolution.
9	71353	Enteritidis PT6a	A	0.060	42/M	New HIV diagnosis, CD4 count 7 × 10^6^ cells/L	No	Fever, cough	Ceftriaxone, co-trimoxazole	Also *Pneumocystis* pneumonia. Resolution.
10	76146	Enteritidis PT1	Pansusceptible	0.060	46/M	Interferon treatment for chronic HBV infection	No	Collapse	Tazocin	Antimicrobial drug changed to ciprofloxacin. Resolution.
11	72429	Enteritidis PT24	ANxCp_L_	1.000	34/M	HIV, CD4 count 17 × 10^6^/L, TB treatment	Thailand	Fever, cough	Ciprofloxacin	Antimicrobial drug changed to ceftriaxone because of slow response. Resolution.
*PT, phage type; A, ampicillin; C, chloramphenicol; Su, sulfamethoxazole; T, tetracycline, Tm, trimethoprim; TB, tuberculosis; Nx, nalidixic acid; Cp_L_, decreased ciprofloxacin susceptibility; SLE, systemic lupus erythematosus; Cro, ceftriaxone; DM, diabetes mellitus; HBV, hepatitis B virus**.**										
